# Ecology of Caribbean Sponges: Are Top-Down or Bottom-Up Processes More Important?

**DOI:** 10.1371/journal.pone.0079799

**Published:** 2013-11-11

**Authors:** Michael P. Lesser, Marc Slattery

**Affiliations:** 1 Department of Molecular, Cellular and Biomedical Sciences, University of New Hampshire, Durham, New Hampshire, United States of America; 2 Department of Pharmacognosy, University of Mississippi, University, Mississippi, United States of America; University of Genova, Italy, Italy

## Abstract

Benthic-pelagic coupling and the role of bottom-up versus top-down processes are recognized as having a major impact on the structure of marine communities. While the roles of bottom-up processes are better appreciated they are still viewed as principally affecting the outcome of top-down processes. Sponges on coral reefs are important members of the benthic community and provide a critically important functional linkage between water-column productivity and the benthos. As active suspension feeders sponges utilize the abundant autotrophic and heterotrophic picoplankton in the water column. As a result sponges across the Caribbean basin exhibit a consistent and significant pattern of greater biomass, tube extension rate, and species numbers with increasing depth. Likewise, the abundance of their food supply also increases along a depth gradient. Using experimental manipulations it has recently been reported that predation is the primary determinant of sponge community structure. Here we provide data showing that the size and growth of the sponge *Callyspongia vaginalis* are significantly affected by food availability. Sponges increased in size and tube extension rate with increasing depth down to 46 m, while simultaneously exposed to the full range of potential spongivores at all depths. Additionally, we point out important flaws in the experimental design used to demonstrate the role of predation and suggest that a resolution of this important question will require well-controlled, multi-factorial experiments to examine the independent and interactive effects of predation and food abundance on the ecology of sponges.

## Introduction

Sponges are ecologically and functionally important members of the benthic community on coral reefs [1]–[6]. In the Caribbean over 80 species have been recorded on reefs in the Florida Keys [7] and close to 300 species have been identified on Bahamian reefs [8]. In addition to their efficient filtering of seawater during feeding [9]–[11], sponges provide essential reef ecosystem functions such as providing habitat for numerous reef species including fish, brittle stars, and shrimp [12], [13]. It is also increasingly recognized that sponges are crucial members of benthic food webs because of their ability to couple water column productivity with the secondary productivity of benthic communities [11], [14]–[16].

Many mobile predators consume sponges (e.g., sea stars, fish, sea turtles), and spongivores can influence competitive interactions between sponges and corals [17]. Sponges have been proposed to be generally well defended against predation by both physical (i.e., spicules) and chemical means [18]–[21]; but see Thoms and Schupp [22] regarding the experimental difficulties of demonstrating chemical defenses in sponges. While it has been shown that cryptic or mangrove sponge species may be limited to specific refugia by spongivores [18], [23]–[25], sponges throughout the Caribbean show a repeatable pattern of increasing biomass and diversity with depth to 150 m [7], [8], [26]–[31].

There is continuing interest in the relative importance of bottom-up versus top-down processes in structuring marine communities generally [32], [34], and coral reef sponge communities in particular [6], [24]. Many studies examining the bottom-up, top-down dichotomy have been conducted on suspension feeding invertebrates in rocky intertidal and subtidal habitats [35]. These studies have generally supported the importance of bottom-up and top-down processes, and the interaction of the two, on the structure of these communities [33], [34]. For sponges on coral reefs, factors that change with depth, such as macro-scale flow velocities, solar irradiance, food supply and water temperature, have all been shown to influence sponge biology and ecology [1], [2], [6], [7], [11], [36]–[38]. Recently, a paper by Pawlik et al. [39] reported on the growth rates of several species of sponge and concluded that rather than bottom-up processes (i.e., food supply) being a primary determinant of sponge growth and abundance, predation, mediated by the presence or absence of chemical defenses, is solely responsible for controlling the ecology of sponge communities. Here we present a natural field experiment *sensu* Diamond [40] demonstrating the importance of food supply in the ecology of *C. vaginalis*, one of the species used in Pawlik et al. [39], and compare this study with studies by Lesser [11], Trussell et al. [15] and Pawlik et al. [39] on *C. vaginalis*. In addition, we discuss in detail the experimental design flaws of Pawlik et al. [39] that make it difficult to come to the conclusions that predation alone structures sponge communities. We do so without any *a priori* dismissal of the potential role that predation, competition or food supply has alone, or together, on the ecology of this species, and on sponges generally.

## Materials and Methods

### Ethics Statement

No endangered or protected species were involved in this field study. No vertebrates were collected in this study; surveys were through visual census only. No “taking” of any flora or fauna was conducted except for samples of seawater. This study was conducted under a research license from Belize Fisheries, which was obtained by the Smithsonian Institution's Caribbean Coral Reef Ecosystems Program.

### Study Site

*Callyspongia vaginalis* Lamarck is a common tubular morphology sponge exhibiting a wide bathymetric and geographic distribution in the Caribbean. Sponge size frequency distributions, picoplankton availability, feeding, tube extension rates and assessments of the total number of sponge species and individual sponge species numbers were conducted at Carrie Bow Cay, Belize (16° 48.16′ N, 88° 04.94′ W) from 3 to 46 m between 2000 and 2001.

### Abundance, Size Frequency and Tube Extension Rates

Transects at 3, 10, 17, 23, 30 and 46 m were conducted by placing a 30 m transect tape parallel to the reef at each depth. Along each transect ten 1.0 m^2^ quadrats were randomly placed and all sponges within the quadrat were enumerated and identified to species where possible. The mean ± SE number of sponges, and number of sponge species, per m^2^ were calculated. Tube length was measured to the nearest 0.1 cm on sponges (N = 10) at each depth, and tubes (N = 3) on individual sponges (N = 10) were tagged 1.0 cm below the lip of the osculum at each depth. Sponges were then left undisturbed for one year when all tagged sponges were re-measured, and tube extension rates (cm mo^−1^) were calculated by subtracting 1 cm from the total change in tube length. In contrast to Pawlik et al. [39], these sponges were not caged so this growth data actually represents the most ecologically relevant condition (i.e., net growth in the presence of both biotic and abiotic factors) although we cannot directly separate the effects of food supply from predation.

### Food Availability and Feeding

Sponge pumping was assessed using sodium fluorescein dye. A small amount of dye (∼1 ml) was injected into the exterior wall of a tube at the base of the sponge (N = 3) with a syringe, and the time it took for the dye to move over a known distance was measured and used to estimate pumping velocity, at a resolution of 0.5 cm s^−1^. The diameter of each osculum was directly measured after dye ejection, and its area estimated using the formula for the surface area of a circle or ellipse. Volume flow rate was estimated by multiplying pumping rate by the cross-sectional area of the osculum. Volume flux was then computed by multiplying ejection speed by the cross-sectional area. This computation assumes plug flow rather than laminar flow coming out of the tube [15]. Careful examination of the dye fronts indicated that plug flow was the better approximation.

After pumping measurements were determined for the same sponges (N = 3) at each depth, ambient seawater was collected approximately 5 cm from the sponge. Matched samples of the excurrent flow were collected from approximately 3 cm inside the osculum of each sponge. Both ambient and excurrent samples were collected with 10 ml Vacutainer® syringes. All samples were fixed at a final concentration of 0.5% electron microscopy grade glutaraldehyde in filtered (0.2) seawater and initially frozen at 0°C for transportation on dry ice to the University of New Hampshire. Samples were then sent frozen to the Bigelow Laboratory for Ocean Sciences Flow Cytometry Facility where they were stored in liquid nitrogen until analysis as described previously [11], [41]. Briefly, each sample was analyzed for cell abundances using a Becton Dickinson FACScan flow cytometer equipped with a 15 mW, 488 nm, air-cooled Argon ion laser. Simultaneous measurements of forward light scatter (FSC, relative size), 90 degree light scatter (SSC), chlorophyll fluorescence (>650 nm), and phycoerythrin fluorescence (560–590 nm) were made on all samples. Differentiation of cyanobacteria from prochlorophytes was based on the presence of phycoerythrin fluorescence. Cell abundance of the heterotrophic bacteria was determined by the use of PicoGreen (Molecular Probes), a dsDNA specific dye, which stains all bacteria (emission fluorescence 515–525 nm). Subtraction of the chl *a* containing picoplankton from total bacteria yielded the heterotrophic bacterial component of the community.

Food availability was separated into cyanobacteria, prochlorophytes, phytoplankton, heterotrophic bacteria, and then total cells. Filtration efficiency was calculated as 1 – (concentration of cells in the ex-current stream/ambient concentration of cells). Cells filtered per second were then computed by multiplying filtration efficiency (dimensionless) x volume flow rate (ml s^−1^) x ambient concentration (cells ml^−1^). All filtered cells were converted to carbon equivalents using the following conversions; heterotrophic bacteria: 20 fg C cell^−1^ [42], *Prochlorococcus*: 53 fg C cell^−1^ [43] and *Synechococcus*: 470 fg C cell^−1^ [44]. The total carbon acquired sponge^−1^ d^−1^ was then converted to energetic units (J s^−1^) using a conversion factor of 1 mg C = 23.03 Joules, assuming an RQ = 1.0 which is appropriate for ammonotelic animals such as sponges [45].

### Spongivores

The diversity and abundance of spongivorous fishes was assessed on replicate 2×10 m band transects at three depths (10, 30, and 46 m) on the fore-reef of Carrie Bow Cay (n = 5 transects per depth). Since spongivores tend to be home ranging [46], on subsequent dives we were able to collect dietary data from replicate individuals (n = 5 from each depth) during 5 min observation periods of each fish. Specifically, we recorded the percent of bites on sponges during the observation period, the total number of bites on sponges during the time period, the number of bites on the same individual, and the number of sponges that each fish species grazed. In addition, the 3-dimensional cover ( =  volume) and identity of sponges within randomly-assigned replicate (n = 5) 1 m^2^ quadrats on each of the transects was assessed. From this data, we were able to determine the ranked abundance of the resident sponges relative to feeding preference (i.e., the ranked bites on specific species).

### Statistical Analysis

Abundance, size frequency, tube extension rates, food availability and spongivore observations were analyzed using one-way ANOVAs at a significance level of 5%, using depth as a fixed factor. No unequal variances were detected using the F_max_ test, and when significant treatment effects were detected individual treatment differences were assessed using the Tukeys HSD multiple comparison test. Where appropriate, ratios and percentages were arcsin or log transformed for analysis and back transformed for presentation. Because the feeding rate of *Callyspongia vaginalis* scaled linearly with sponge size in a previous study [11] an ANCOVA was run on all feeding data with sponge size as a covariate.

## Results

**Figure 1:**
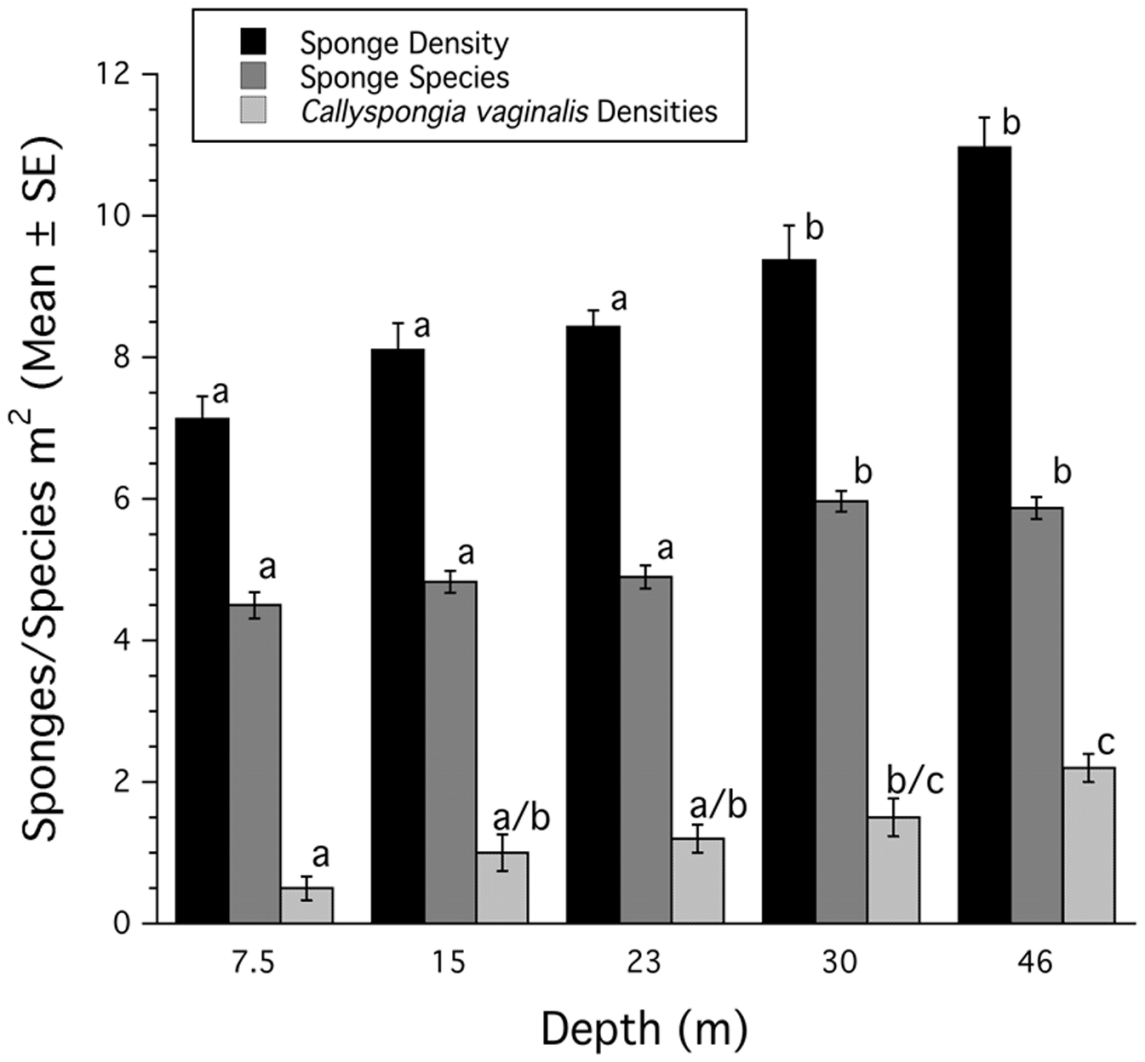
Sponge densities and diversity on Carrie Bow Cay, Belize (m^2^, mean ± SE). Density of sponges, number of sponge species and density of *Callyspongia vaginalis* from different depths. Common superscripts indicate groups not significantly different from each other.

At Carrie Bow Cay sponge density increases significantly (ANOVA: p<0.0001) with increasing depth, as does the number of species (ANOVA: p<0.0001, Fig. 1). Sponge density and species number were significantly different between shallower (7.5–23 m) and deeper (30–46 m) depths (Tukeys HSD: p<0.05). Additionally, the density of *Callyspongia vaginalis* increased significantly with depth (ANOVA: p<0.0001, Fig. 1) with significant differences between the shallowest and deepest depths, and the middle depths exhibiting overlapping differences (Tukeys HSD p<0.05).

**Figure 2:**
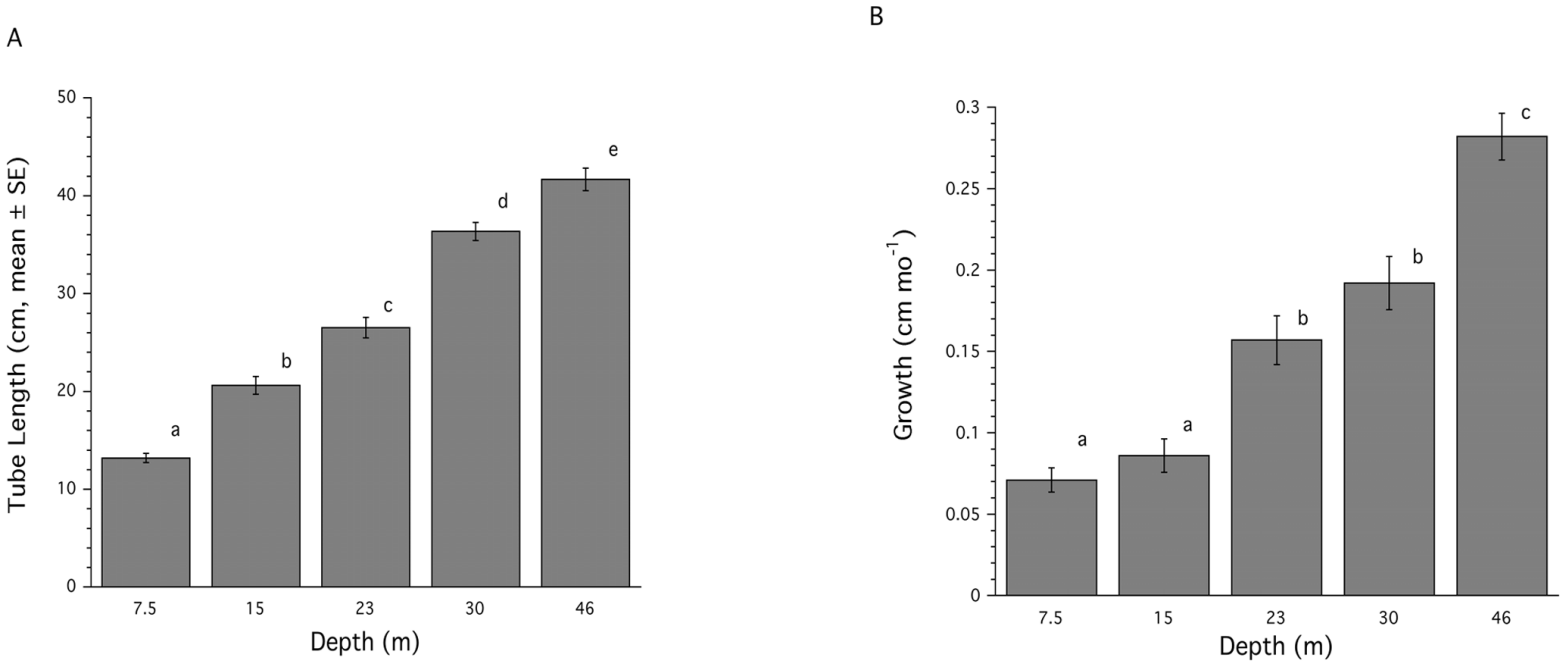
Tube length and growth for *Callyspongia vaginalis* on Carrie Bow Cay, Belize. Tube length (cm, mean ± SE) (A). Growth over a one-year period (cm, mean ± SE) (B). Common superscripts indicate groups not significantly different from each other.

Sponge size, measured as tube length, showed a significant (ANOVA: p<0.0001) effect of depth for *Callyspongia vaginalis* with larger sponges found at deeper depths (Fig. 2 a). Multiple comparison testing showed that tube length at each depth was significantly different (Tukeys HSD: p<0.05) from each other (Fig. 2 a). Measurements of tube extension rates (cm mo^−1^) showed a significant effect (ANOVA: p<0.0001) of depth for *Callyspongia vaginalis* (Fig. 2 b). Multiple comparison tests showed that sponges from the deeper depths (23–46 m) grew significantly faster (Tukeys HSD: p<0.05) than sponges from shallow depths, and sponges at 46 m grew faster than sponges at all other depths (Fig. 2 b).

**Figure 3:**
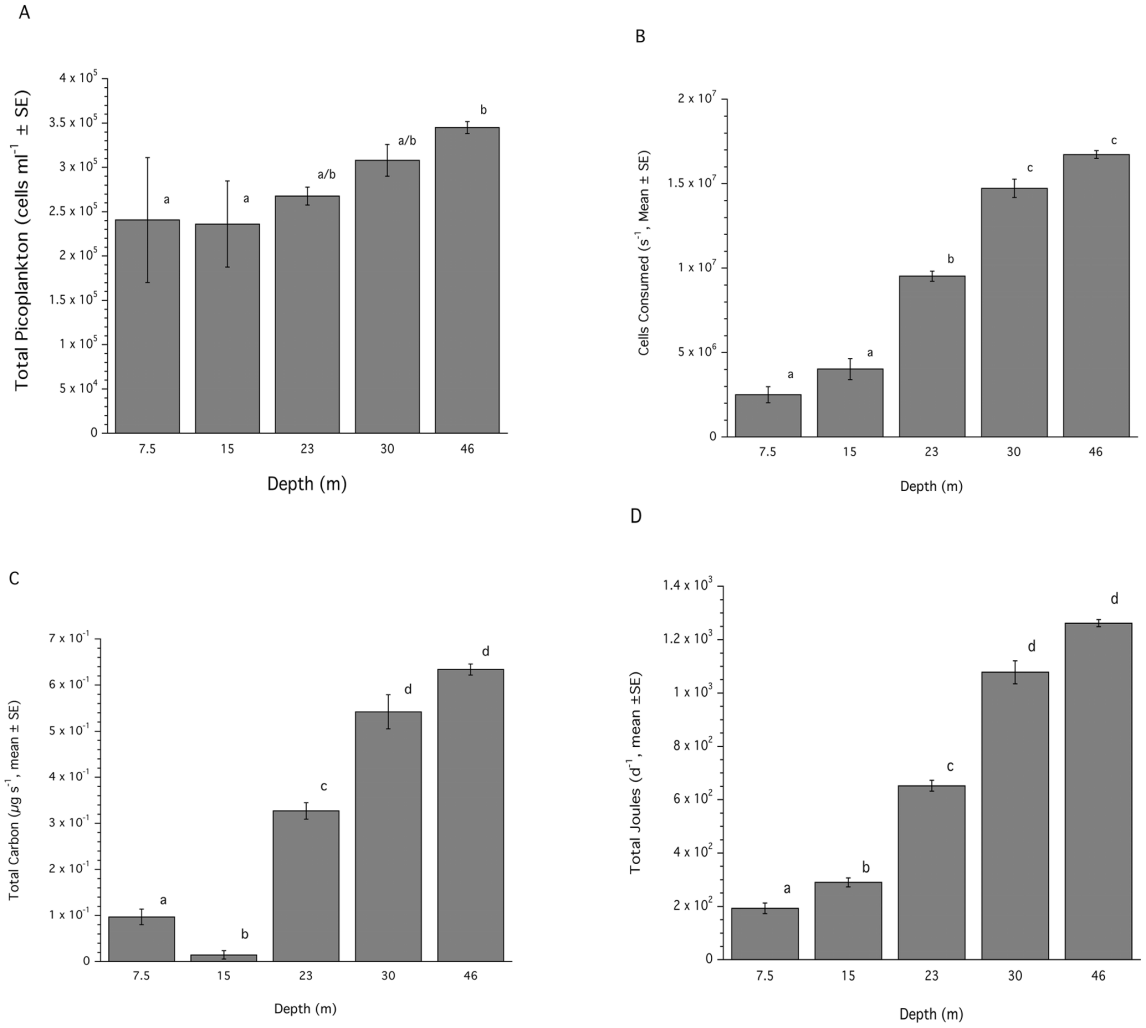
Picoplankton availability and feeding for *Callyspongia vaginalis* on Carrie Bow Cay, Belize. Depth-dependent concentrations of total picoplankton (A). Instantaneous total cells consumed per individual at each depth (B). Instantaneous intake of total particulate carbon per individual (C). Daily intake of total energy (J) per individual (D). Common superscripts indicate groups not significantly different from each other.

Total food availability for *C. vaginalis* increased significantly (ANOVA: p<0.031) with depth (Fig. 3 a) with the highest concentration of picoplankton found at 46 m (Tukeys HSD: P<0.05). As observed in previous studies [11], the density of cyanobacteria within the picoplankton decreased while both the prochlorophytes and heterotrophic bacteria increased with depth (data not shown). The calculated percentage clearance for *C. vaginalis* was also significantly different with depth (ANOVA: p<0.0013), and ranged from 53% at 7.5 m to 84% at 46 m. The clearance rate at the shallowest depth (7.5 m) was significantly different (Tukeys HSD: p<0.05) from all other depths that were not significantly different from each other (data not shown). Unlike previous observations from the Florida keys where only two depths were analyzed [11] the ANCOVA analysis for the effects of depth, with tube length as a covariate, on feeding rates as well as carbon and energy acquisition was not significant (ANCOVA: p<0.05) and was not considered further. Combining the available food with percentage clearance and the volume flux calculations showed that *C. vaginalis* consumed significantly more food with increasing depth (ANOVA: p<0.0001), and acquired more carbon as particulate organic carbon (ANOVA: p<0.0001) and more energy (J d^−1^; ANOVA: p<0.0001), based on an assumption of constant pumping rate and feeding (Fig. 3 b). In all cases multiple comparison tests showed that sponges at shallower depths had significantly lower rates of feeding, total carbon (Fig 3 c) and total energy acquisition (Fig. 3 d) when compared to deeper depths (Tukeys HSD: P<0.05).

There were no significant differences in spongivorous fish densities between the shallow reefs (10 and 30 m) and the deep reef (46 m), with the exception of spadefish that were not observed shallower than 39 m (Table 1; ANOVA: p = 0.0326). The percent of sponges in spongivorous fish diets was consistent across the depth gradient (ANOVA: p = 0.0897), although the number of sponge species in the diet of spongivores increased significantly with depth (ANOVA: p<0.0001). Finally, the number of bites per unit time was consistent across the depth gradient (ANOVA: p = 0.1121), but spongivores on the deeper reef exhibited significantly more focal bites on individual sponges than did their conspecifics on the shallow reefs (ANOVA: p<0.0001). *Callyspongia vaginalis* was the 12^th^ and 23^rd^ most common sponge, by volume, on the shallow and deep reefs, respectively (data not shown). Additionally, *C. vaginalis* represented the 19^th^ and 24^th^ most common prey species on shallow and deep reefs respectively, based on focal bites.

**Table 1:** Depth-specific spongivore densities and their diet on Carrie Bow Cay, Belize.

Spongivore	Density: 10–30 m (2×10 m)^−1^ N = 10	Density: 46 m (2×10 m)^−1^ N = 5	Dietary Breadth (% bites on sponges) [# sponge species eaten]	Bites (15 min)^−1^	Bites (individual)^−1^
CHAETODONTIDAE					
*Chaetodipterus jaber*	0	2.6±1.6	na/31.4±4.5 [na/10.4±1.3]	0/10.2±3.1	0/4.2±0.9
MONOCANTHIDAE					
*Cantherhines macrocerus*	0.2±0.1	0.6±0.4	66.7±3.6/77.2±3.7 [7.9±0.5/10.2±1.3]	21.3±2.7/18.6±2.9	1.6±0.3/5.0±1.0
POMACANTHIDAE					
*Holocanthus ciliarus*	0.3±0.2	0.4±0.4	94.5±1.7/98.4±0.9 [24.7±1.0/35.2±2.7]	12.2±1.4/13.0±1.9	2.6±0.4/4.0±0.8
*Holocanthus tricolor*	0.6±0.3	0.4±0.2	92.8±1.5/97.0±1.5 [21.5±1.2/28.8±0.9]	17.8±1.2/19.0±2.1	1.6±0.2/10.2±1.5
*Pomacanthus arcuatus*	0.5±0.3	0.4±0.4	70.7±2.5/74.8±2.7 [21.2±1.3/28.8±0.9]	12.4±0.9/14.2±1.9	1.2±0.1/6.0±0.7
*Pomacanthus paru*	0.4±0.2	0.2±0.2	76.5±2.5/73.4±2.9 [19.7±0.7/30.4±1.4]	14.9±0.8/14.8±2.2	1.2±0.1/6.2±1.2
TETRODONTIDAE					
*Canthigaster rostrata*	4.8±1.5	4.4±1.8	20.3±2.4/45.4±2.1 [11.2±0.8/28.0±0.7]	47.9±12.1/48.8±9.1	1.9±0.3/12.8±2.9

Notes: Data presented are the mean number of fish ± SE per 2×10 m band transects (n = 5 replicates at each of three depths: 10 m, 30 m and 46 m). Percent of bites on sponges for each fish species are based on 15 min bite count observations of five fish on the shallow- and deep- reefs (na  =  not applicable: no fish observed at depth); the number of sponge species sampled during that period are included in parentheses. The average number of bites per 15 min observation period, and the average number of focal bites on individual sponges are recorded. In all cases where numbers are separated by a hash (“/”), the first number refers to the shallow depths and the second number refers to the deep depth.

## Discussion

In tropical ecosystems neritic and pelagic productivity is dominated by picoplankton, including both photoautotrophic and heterotrophic bacteria [11], [47], [48]. The small size of these picoplankton (1.0 µm or less) makes them an ideal food resource for sponges that are active suspension feeders and consume a large fraction of these microbes [10], [11], [49]. Caribbean sponges such as *Callyspongia vaginalis*, *Agelas conifera*, and *Aplysina fistularis* have been shown to acquire their food heterotrophically [11], [15], unlike their photoautotrophic counterparts typical of Pacific reefs such as the Great Barrier Reef [1], [2].

Here we show that the bathymetric pattern of picoplankton distribution for cyanobacteria (*Synechococcus* sp.), prochlorophytes and heterotrophic bacteria (likely including various chemoautotrophs and Archaea) is similar to what has been previously reported for the waters adjacent to coral reefs across the Caribbean [11]. The carbon and energy acquired by sponges from these planktonic resources are substantial and result in a consistent and repeatable pattern of increasing sponge size and growth rates for both chemically defended and undefended species with increasing depth ([11], this study). The natural experiment described here used a chemically undefended sponge that is fully exposed to equivalent numbers of spongivores, specifically fishes [30], [39], [50], at depths up to 46 m, with no evidence for depth dependent differences in spongivory on this species. If anything, since spongivorous fishes are likely to follow their food supply [6] one would predict higher rates of sponge biomass removal by these predators with increasing depth that is not evident in this study or others [e.g., 11]. We did observe a greater dietary breadth at depth, and more focal bites on individual sponges, but this apparently had no effect on growth rates of sponges at depths greater than 30 m.

For *Callyspongia vaginalis* in particular, a well-controlled reciprocal transplant experiment was conducted on Conch Reef in the Florida Keys [15] the same reef where Pawlik et al. [39] conducted their studies. In that study *C. vaginalis* from the deep reef grew significantly (27%) more than conspecifics from the shallow habitat. To examine the effect of depth and the genetic or environmental basis of habitat-specific variation in sponge growth, Trussell et al. [15] conducted a reciprocal transplant experiment between deep (25 m) and shallow reefs (12 m). Sponges returned to their native site served as transplant controls (e.g., shallow —> shallow). A significant depth effect on sponge growth indicated that: 1) deep control sponges exhibited significantly higher growth rates than both shallow sponge controls and deep sponges transplanted to the shallow site, 2) there were no significant differences in growth between deep and shallow sponges maintained at the deep site, and 3) shallow sponges transplanted to the deep site showed only a trend of increased growth (0.36±0.11 cm) over their controls (0.20±0.11 cm). These data indicate that deep sponges generally grow faster than shallow sponges but are not able to maintain their high growth rates when transplanted to the shallow habitat. In addition, the large differences in growth observed between both control groups were not apparent between shallow sponges transplanted to the deep site and deep site controls. Hence, shallow sponges placed in the deep habitat and deep controls exhibited comparable growth rates. Moreover, without having tested directly the effects of predation, all of these results occurred while being exposed to the full suite of spongivores.

These results are also supported by simultaneous assessment of food availability, feeding and energetic budgets for sponges at each depth and the reciprocal transplants [15]. Deep reef habitats at Conch Reef are more conducive to higher growth rates than the shallow reef, which is consistent with the larger overall population size structure of sponge populations in the deep vs. shallow reefs on Conch Reef [11]. Moreover, the shift in the growth rates of deep reef sponges between the two habitats and the trend for shallow sponges suggests a phenotypically plastic response to different environments, rather than genetically different populations which has since been quantified for *C. vaginalis* [51]. The work on sponges at Conch Reef by Lesser [11] and Trussell et al. [15] is also consistent with transplant experiments done by Leichter et al. [52] that showed higher growth rates at 30 m versus 15 m for the coral *Madracis mirabilis* on Conch Reef. Additionally, long-term population studies on the giant barrel sponge *Xestospongia muta*, a sponge that exhibits highly variable chemical defenses [20], showed that sponge densities increased 2–5 times faster at 30 m compared to 10 m and 20 m, while sponge size frequency distributions shifted to smaller sponge sizes across all depths at Conch Reef [53]. McMurray et al. [53] attributed increases in sponge densities to episodic recruitment events and subsequent survival but did not explain the depth dependent density differences. If recruitment potential is the same over the depth range of 10–30 m, and the densities of spongivores are equivalent across depths at this location [39], then we would hypothesize recruitment survival could be explained by access to greater food resources at 30 m.

There are several flaws with the experimental design of Pawlik et al. [39] that reduce the impact of their conclusion regarding control by spongivorous fish for Caribbean sponge communities. First, in their *C. vaginalis* experiment Pawlik et al. [39] lost an entire treatment group which necessitated the use of multiple *t*-tests rather than the planned multifactorial ANOVA. However, the authors appear to use multiple *t*-tests without correcting the comparison-wise *p* value to control for the experiment-wise α of 0.05 (i.e., the 30 m uncaged treatment group was used in more than one comparison) and avoid making a Type I error [54]. In fact, it appears that correcting the *p* value for the number of multiple comparisons to an α of 0.025 shows that their results for caging effects (*p* = 0.0486) would not be significant. Also, there is no explanation for running a one-tailed *t*-test. Prior data in fact already shows greater growth at deeper depths [11] which *a priori* should have led the authors to conduct a two-tailed test to test for the possibility of greater growth in either shallow or deeper depths and the required doubling of those *p* values. Second, not having cage controls is a clear experimental design flaw, despite the report of no cage effects from Leong and Pawlik [55] where similar problems with *post hoc* multiple comparison analyses also occur; a study that itself did not report the cage control data. Cages will affect the hydrodynamics outside and inside the cage and therefore the flow speed and residency time of the water within the cage [56] with potential effects on food availability and sponge metabolism. Another underappreciated effect of caging would be a significant reduction in flow-induced feeding by sponges [57], [58]. Furthermore, cages could also affect light levels within these structures (i.e., they will act as neutral density filters) that could negatively affect sponges in Pawlik et al. [39] that contain photoautotrophic symbionts (i.e., *Aplysina cauliformis*) [59], [60]. There were, however, clearly no effects of cages or depth on the growth of *Amphimedon compressa and Aplysina cauliformis* and, not withstanding the lack of cage controls, this suggests that food supply, predation, and in the case of *A. cauliformis* light, did not significantly affect the growth of these sponges in these experiments so broad conclusions about the role of predation appear unsupported in this case. But controlling for cage effects in each experiment, even if the results agree with *a priori* anticipated outcomes, is required and when conducted appropriately can be very informative regarding the role of fish predation [61], [62]. Third, sponges were collected from “10–30 m” and then divided into depth treatment groups (15 m and 30 m) with no regard to their prior history at the depths of collection. The appropriate design is to conduct reciprocal transplants, with disturbance controls [15], [63], that match experimental depths with the sponge collection depths [15], [64] such that any genetic, or prior environmental history differences or disturbance effects can be detected and accounted for. Finally, the assessment of sponge growth by Pawlik et al. [39] is by buoyant weight but the results are described on a percentage basis that has the potential to mask what are actually very small increases in biomass. While we appreciate the importance of volumetric parameters (i.e., wall-thickening) as a component of growth (e.g., [5]), in the specific case of *C. vaginalis* previous work has shown that both volume and tube length, in absolute units, increases with depth in *C. vaginalis* [11], [15].

We also disagree with Pawlik et al. [39] that Conch Reef is typical of the coral reefs around the Caribbean basin. Pawlik et al. [39] note that the unique oceanography at Conch Reef (i.e., internal waves that drive a “plankton pump” [52]), should provide the strongest signal in the Caribbean basin for bottom-up processes, but the feeding and physiological experiments by Lesser [11] and Tussell et al. [15] were actually conducted during times when internal waves were absent. We do, however, appreciate how internal waves integrated annually would increase productivity and food availability to deeper coral reef communities *sensu* Leichter et al. [52], [65], [66] and effect long-term growth rates. It is therefore important to see similar results for *C. vaginalis* reported here from Carrie Bow Cay, Belize; the site is a Caribbean coral reef system that is not significantly affected, in terms of frequency or amplitude [67], by an internal wave driven plankton pump but there is a significant increase in picoplankton abundance with increasing depth, and the sponges on this reef are exposed to abundances of spongivores that are similar to what is found on Conch Reef ([6], Table 1).

Pawlik et al. [39] go on to argue that Conch Reef and other reefs throughout the Caribbean basin have a similar diversity of sponges and spongivores. In fact, sponge diversity on Conch Reef may be the lowest in the region (e.g., [7] vs. [8]), and even if diversity is similar function can be quite different [68]. Also, while spongivore diversity is much more similar across the region at depths up to 30 m, except for parrotfishes which arguably are not important spongivores [6], [50], [69], differences in spongivore abundance and diversity can have significant impacts on community structure, and likely function but mostly for reefs >50 m in depth ([30], Slattery and Lesser unpublished data).

When exposed to spongivorous fishes, *Callyspongia vaginalis*, a widely distributed and chemically undefended sponge ([20], Slattery unpublished data), maintains greater biomass and grows faster in proportion to the abundance of picoplankton ([11], [15], this study). This abundant sponge is also of poor nutritional quality [70], and sponge nutritional quality has been shown to be more important than chemical defenses in determining whether fish consume different species of sponges [71]. Despite claims that differential predation, mediated by chemical defenses, is the major process controlling the ecology of sponges [19], [39], this view is not universally supported [6], [50] and the data on food availability and growth, differences in sponge nutritional quality, competition and disease all support roles for other processes in the ecology of sponges [5], [72]. In fact, our data (Table 1) indicates that spongivory is consistent across depths from 10 to 46 m at Carrie Bow Cay, but the greater sponge biodiversity and biomass on deeper reefs has resulted in a more opportunistic feeding strategy that includes more sponge species as food and potentially less time searching for prey (i.e., more focal bites per individual). It is also possible that the latter behavior implies that these sponges are less defended than their shallow reef counterparts (e.g., Slattery et al. unpublished data), although this reinforces our point that predation has limited control on sponge growth rates on deep reefs since any tissue loss is compensated by enhanced food supply for either defended or undefended sponges [11]. It is interesting to note that based on their rank availability (12^th^) and their ranking as a prey species (19^th^) shallow reef *C. vaginalis* may have some feeding deterrent characteristics (i.e., chemical defenses and/or lowered nutritional content). In contrast, deep reef *C. vaginalis* are consumed at a level consistent with their availability in the population.

Understanding the ecology of sponges is more important than ever as there is increasing awareness that some tropical reefs could undergo a phase transition to sponge dominated reefs [73]. There is some evidence from long-term data that this is already happening [74], even in the presence of spongivorous fishes [75]. But evidence of sponge declines, putatively from disease outbreaks, also exist [5], [76], and a recent cold-water event in the Florida keys resulted in significant decreases in both coral and sponge cover [77]. Importantly, Caribbean wide declines in coral reef fish have also been reported with significant declines in “invertivores” that include well-known spongivorous species [78]. Paddock et al. [78] conducted a meta-analysis of data from 1955–2007 and found that the most significant decline in fish populations occurred during 1996–2007. This begs the following question; if you believe, or can show, that spongivory by fishes is the primary process controlling sponge populations, is there a “tipping point” for sponge populations where predation pressure becomes so low that abiotic factors (e.g., food supply) become more important over time [79]? Additionally, the Caribbean wide invasion by lionfish has further reduced fish populations since 2005, including spongivores, on both shallow [80] and mesophotic [30] reefs.

Pawlik et al. [39], despite claims to the contrary, did not directly test the role of bottom-up factors, or food supply and feeding, on the ecology of their target sponges. Similarly, Lesser [11], Trussell et al. [15] and the data presented here did not directly test the role of predation on sponges. This is reminiscent of early discussions on the role of nutrients versus herbivory on the phase transition of reefs from coral- to algal-dominated reefs [75], [81]-[84]. No one had yet carried out the multi-factor, multi-level experiments required to tease apart the respective roles of nutrients versus herbivory, and their interaction, in these phase transitions (*sensu* [85]). When that was finally accomplished it was found that herbivory controls algal production, but that nutrients can play an important role under certain circumstances [86]. In a similar manner, no one has yet conducted carefully controlled experiments that examine the independent and interactive effects of predation and food supply on the ecology of sponges. To do this requires a detailed and controlled experimental approach carried out on sponges under varying levels of food supply and predation pressure. We predict that, similar to algal phase shifts on coral reefs [75], [84], [87], the controlling factors will be much more complex than either predation or food levels in isolation.

## Acknowledgments

We thank Deborah Gochfeld for constructive comments on the manuscript.
